# Components of Service Delivery Models of Care for the Detection, Care and Management of Visual Impairment for Adults and Children with Acquired Brain Injury: A Scoping Review Protocol

**DOI:** 10.22599/bioj.532

**Published:** 2026-02-10

**Authors:** Lauren R. Hepworth, Ffion Curtis, Michelle Maden, Andrew Hill, Brinton Helliwell, Charlotte Croft, Ruaraidh Hill, John Ravenscroft, Shelley Robinson, Catrin Tudur Smith, Cathy Williams, Fiona J. Rowe

**Affiliations:** 1 University of Liverpool, UK; 2 Mersey and West Lancashire Teaching Hospitals NHS Trust, UK; 3 VISable, Liverpool, UK; 4 University of Edinburgh, UK; 5 Kent County Council, UK; 6 University of Bristol, UK

**Keywords:** acquired brain injury, ABI, visual impairment, evidence synthesis, scoping review, health services

## Abstract

**Introduction:**

Vision problems are common in patients with acquired brain injury (ABI). Inpatient eye care is ad hoc in the UK, and once patients with ABI are discharged from hospital care, community eye care provision is typically non-standardised and frequently leads to substantial unmet needs and health inequalities for patients.

**Objective:**

The objective of this scoping review is to identify and describe the components of existing service delivery models of care in relation to the detection, care and management of visual impairment for adults and children with ABI. This is a protocol; no results will be published in this manuscript.

**Methods:**

We will search multiple databases to include MEDLINE, CINAHL, Embase, APA PsycINFO and The Cochrane Library for English language publications from the Organisation for Economic Co-operation and Development (OECD) countries. Supplementary searches will also be conducted to locate grey literature. We will include any study that describes or assesses service delivery for adults and children with visual impairment following ABI in health and social care and education settings.

**Stakeholder Engagement:**

We consulted with a broad range of stakeholders, including subject experts from health and education sectors and patient and public representatives during the planning, and will continue the same during the conduct, reporting and dissemination of this scoping review.

## Introduction

Acquired brain injury (ABI) is defined as being caused by acute events occurring after the peri-natal period (> 1 month old) inclusive of focal injury (e.g., stroke, tumours), traumatic injury and central nervous system infections, but not genetic, congenital, pre- or peri-natal causes. In the UK, there were 335,409 hospital admissions for ABI in 2023–2024 (Headway, 2025).

Visual impairment is common in individuals following an ABI, with estimated prevalences ranging from 54% to 73% (Greenwald, Kapoor and Singh, 2012; Rowe *et al*., 2019). Post-ABI visual impairment is often complex and multifaceted, causing significant challenges to both individuals and wider society (Armstrong, 2018; Falkenberg *et al*., 2020; Hepworth and Rowe, 2016). Visual impairments are wide-ranging, including reduced central vision, visual field defects, oculomotor defects, binocular vision dysfunction, light sensitivity and visual perception deficits (Greenwald, Kapoor and Singh, 2012; Merezhinskaya *et al*., 2019; Rowe *et al*., 2020).

Sight was found to be the most valued sense in a UK survey of the general public, with participants choosing a shorter life with perfect health versus one with visual impairment (Enoch *et al*., 2019). Sight is a key requirement for many activities of daily living. However, visual impairment is not always obvious and patients may not report visual symptoms; therefore, detection relies on specific tests appropriate to age and ability (Armstrong, 2018; Hepworth *et al*., 2021; Lindstedt *et al*., 2019). It is therefore crucial to have appropriate, accurate and early patient assessments and high-quality evidence-based eye care provision delivered in appropriate care settings (Rowe *et al*., 2025; Schow *et al*., 2024).

Ideally, for ABI, service provision is needs-driven and meets the requirements of the individual. For ABI, this warrants early assessment, management and support, and is often first needed as part of inpatient bedside care. Once patients with ABI are discharged from hospital care, community eye care and liaison with community and social care services is typically non-standardised and frequently leads to substantial unmet needs for patients. Whilst hospital-based assessment and management are well-documented, models of care can vary widely, and inpatient eye care is ad hoc in the UK (Hepworth and Rowe, 2019). There also are gaps in high-quality evidence relating to the organisation of health care to detect and manage these conditions, as well as how to better integrate care across National Health Service (NHS) and partner services such as social services, education and charity sectors.

The complexities and deficits associated with ABI warrant a comprehensive evaluation and treatment approach provided by a multidisciplinary team consisting of orthoptists, ophthalmologists, optometrists, neurologists, paediatricians, occupational therapists and teachers of learners with visual impairments (including qualified teachers of learners with visual impairment (QTVIs)), among others. Integrated service provision requires diverse groups of professionals situated across a range of organisational settings to work collaboratively to provide care for complex cases (Wehling *et al*., 2024).

This review aligns with four research priorities within neuro-ophthalmology, as set out by the UK clinical eye research strategy—how can the prevention, diagnosis and treatment of neuroinflammation/stroke/intracranial tumours affecting vision be improved, and how can the diagnosis and treatment of traumatic brain injury affecting vision be improved (Bourne *et al*., 2024)? What is needed now is an overview of existing service delivery around the integrated service provision for the detection and management of post-ABI visual impairment to provide a better understanding of current practice.

## Aims and Objectives

This is a protocol; no results will be published in this manuscript. The aim of this scoping review is to identify and map common elements of global ABI service delivery models around the detection and management of visual impairment and collaborative practice, capturing care resources involved to scope potential care costs. This will enable us to systematically map research findings across a body of research evidence that is heterogeneous and/or complex in nature to inform future service delivery in the UK.

### Objectives

- To describe the content, configuration and setting of health and wider care resources involved in services for post-ABI visual impairment,
- To describe the magnitude, frequency and duration of consultations and appointments,
- To describe and map the cost implications,
- To identify and map elements of integrated service provision and service delivery models for post-ABI visual impairment.

A preliminary search of MEDLINE, the Cochrane Database of Systematic Reviews and JBI Evidence Synthesis was conducted and no current or underway systematic reviews or scoping reviews on the topic were identified.

## Methods

The proposed scoping review will be conducted in accordance with the JBI methodology for scoping reviews (Peters *et al*., 2020). This protocol follows the Preferred Reporting Items for Systematic Reviews and Meta-Analyses (PRISMA) reporting guidelines for protocols (Shamseer *et al*., 2015) and scoping reviews (Tricco *et al*., 2018). Patient and public representatives have helped shape the review question and will contribute to the conduct and reporting of this scoping review.

### Identifying relevant studies

Eligibility criteria: A broad range of written sources will be considered to include research studies, systematic reviews, service evaluations, audits, guidelines, quality standards or practice recommendations. Study protocols, conference abstracts, editorials and letters will be excluded.

Population: Adults and children (aged > 1 month old) with visual impairment following ABI, carers or professionals involved in the delivery of services post-ABI visual impairment. We define ABI as being caused by acute events occurring after the peri-natal period (> 1 month old) inclusive of focal injury (e.g., stroke, tumours), traumatic injury and central nervous system infections, but not genetic, congenital, pre- or peri-natal causes.

### Concept

ABI commonly causes visual impairment, which can include multiple vision problems, including reduced central vision, visual field defects, oculomotor defects, binocular vision dysfunction, light sensitivity and visual perception deficits (Greenwald, Kapoor and Singh, 2012; Merezhinskaya *et al*., 2019; Rowe *et al*., 2020). Symptoms of post-ABI visual impairment may not be self-reported due to communication and/or cognitive problems associated with ABI (Hepworth *et al*., 2021). Early assessment, management and support are required to maximise rehabilitation, including return to work, school and activities of enjoyment (Edwards *et al*., 2006; Rowe, 2017; Smith *et al*., 2018).

### Context

Currently, health inequalities exist where, for example, service provision is ad hoc and non-standardised across geographical regions (Hanna, Mercer and Rowe, 2020; Hepworth and Rowe, 2019; Sorbello, 2022). Service delivery models—how services are delivered, organised and how they assess and manage patients with post-ABI visual impairment—will be included. Elements of service delivery models will include (but are not limited to), screening and specialist tests, settings of delivery, staff involvement, timing of assessment and follow-up, referral pathways and clinical and cost outcomes. Primary treatment options for ABI will be excluded.

Studies conducted in hospital, community, educational and home settings will be included. We will include studies from countries with an Organisation for Economic Co-operation and Development (OECD) membership. Any study that describes or evaluates service delivery around the detection or management of post-ABI visual impairment (e.g., systematic reviews, randomised controlled trials, surveys, process evaluations, economic evaluations) will be included.

### Information sources

We will search MEDLINE, CINAHL, Embase, APA PsycINFO, Cost-Effectiveness Analysis (CEA) Registry, International HTA Database, ERIC, British Education Index, Education Research Complete, and The Cochrane Library (CENTRAL and CDSR) from inception onwards, restricted to English language publications only.

Supplementary searches will also be undertaken for a UK-focused search of grey literature such as: Health Management Information Consortium (HMIC), websites of selected relevant organisations—for example, RNIB, The Neurological Alliance, The Brain Charity, etc. We will also conduct targeted Google Scholar, LENS.org and Web of Science searches. A call for evidence will be made to the clinical and educational communities involved in the post-ABI visual impairment care for reports, service evaluations, audits, guidelines, quality standards, practice recommendations and notices of ongoing research.

### Searches

A comprehensive and wide-ranging search will explore services across the spectrum of hospital, community, social care, education and other related settings for post-ABI visual impairment.

An initial exploratory search will be developed in an iterative manner in MEDLINE from keywords relevant to post-ABI visual impairment, identified by the review team (which includes an information specialist), stakeholder group and published topic and methodologically relevant systematic reviews. Keywords will include variations on the following terms: ABI AND visual impairment AND service delivery. Subject headings (e.g., MeSH), free text and advanced search techniques (e.g., truncation, proximity operators) will be used. The initial search for each topic will evolve during discussions with the wider research team and stakeholder group to ensure that the search terms are relevant.

A sample of relevant records will be used to conduct sensitivity analysis on search terms to check the sensitivity of the searches and amend as required. An iterative approach to searching will be undertaken as our understanding of the literature increases. Once the searches are tested and validated in MEDLINE, we will translate them across other sources. No date or study design limitations will be applied to the search strategies, but we will exclude animal studies. In addition, we will contact topic experts to seek unpublished/ongoing studies, check reference lists of included studies and carry out forward citation searching.

### Selection of evidence

Search results will be downloaded into a bibliographic record management system (e.g., EndNote) and de-duplicated before uploading for screening and selection using a review management platform (e.g., Rayyan). Studies will be selected for inclusion through a two-stage process, that is, title/abstract screening followed by full text screening using the predefined explicit criteria. Title/abstract screening will be completed independently by two reviewers to exclude records that do not meet the inclusion criteria and select records that may meet inclusion criteria for further review at the full text stage. Full texts of those selected will be retrieved and independently screened by two reviewers against the inclusion criteria. Any disagreements on eligibility decisions will be resolved through consensus and, if necessary, by discussion with a third reviewer.

The results of the search and the study inclusion process will be reported in full in the final scoping review and presented in a Preferred Reporting Items for Systematic Reviews and Meta-analyses extension for scoping review (PRISMA-ScR) (Tricco *et al*., 2018).

### Data charting

A standardised data extraction form will be developed and piloted. Data to be extracted will be informed by discussions with our stakeholder group. Data relating to study characteristics (e.g., author, date, country, study design), sample (e.g., patient demographics, sample size), models of service delivery (e.g., types of services/test/intervention provided, format, delivery, intensity, frequency, duration, underlying framework or theories, etc.) and equity will be extracted by one reviewer and independently checked for accuracy by a second reviewer.

The data extraction tool will be modified and revised as necessary during the process of extracting data from each included evidence source. Modifications will be detailed in the scoping review. Any disagreements that arise between the reviewers will be resolved through discussion, or with an additional reviewer(s). If appropriate, authors of papers will be contacted to request missing or additional data, where required.

### Data analysis and presentation

A narrative synthesis of the descriptions of service delivery will be reported. We will highlight common elements of what reportedly does and does not work in relation to service delivery models. Statistics on the effects of service delivery models will be extracted as reported, but, where data allows, standardised in evidence tables (noting any calculations). To compare the economic results of individual studies, costs will be converted to 2024 GBP by applying the gross domestic product deflator index and purchasing parities conversion rates using the CCEMG-EPPI cost converter tool (Shemilt, Thomas and Morciano, 2010), and a narrative approach will be used to present these findings. Evidence will be summarised in tables and synthesised narratively (Campbell *et al*., 2020).

Stakeholder Engagement: We will continue to consult with a broad range of stakeholders, including subject experts and patient and public representatives during the planning, conduct, reporting and dissemination of this scoping review. We will have regular meetings and share documents to receive feedback on all review tasks, including the scope of the review, search terms, the type of evidence to include, data presentation, reporting and dissemination planning.

Dissemination: All data in this project will be secondary, gathered through database searching or the call for evidence. We will develop our dissemination strategy with stakeholder input, which will include sharing our findings via social media, peer reviewed publications, existing networks and stakeholder engagement meetings and events.

## Summary

For ABI, early assessment, management and support is essential to maximise rehabilitation potential. Visual impairment post-ABI causes significant challenges for activities of daily living and impacts an individual’s independence and quality of life (Hepworth and Rowe, 2016; Wagener and Kreiger, 2019). Currently, there are gaps in how evidence relating to the assessment and measurement of the psychosocial impact of these conditions is being applied in practice, how the organisation of health care functions to detect and manage these conditions, as well as how to better integrate care across the NHS and partner services such as education, social services, and the charity sector. This scoping review will identify and present much needed information on models of service delivery across the care pathway for individuals with post-ABI visual impairment.
